# Clinical Predictors of *Escherichia coli* Versus *Staphylococcus aureus* Bacteremia at the Emergency Department

**DOI:** 10.3390/antibiotics14070654

**Published:** 2025-06-27

**Authors:** Pariwat Phungoen, Thanat Tangpaisarn, Kittisak Sawanyawisuth

**Affiliations:** 1 Department of Emergency Medicine, Faculty of Medicine, Khon Kaen University, Khon Kaen 40002, Thailand; 2 Department of Medicine, Faculty of Medicine, Khon Kaen University, Khon Kaen 40002, Thailand

**Keywords:** bacteremia, *E. coli*, *S. aureus*, solid organ tumor

## Abstract

**Background:** Bacteremia is a life-threatening condition encountered in the emergency department (ED). *Escherichia coli* and *Staphylococcus aureus* are among the most common pathogens, but early differentiation is challenging. Identifying clinical predictors may help guide empirical treatment while awaiting culture results. **Methods:** This retrospective analytical study included adults aged 18 years or older who presented with bacteremia in the ED between 1 January 2016 and 31 December 2018 and had blood cultures positive for either *S. aureus* or *E. coli*. Clinical predictors of *E. coli* bacteremia were identified using multivariable logistic regression analysis. **Results:** Among 327 patients, 272 (83.2%) had *E. coli* bacteremia. Significant predictors of *E. coli* bacteremia included hypertension (adjusted OR 2.12; 95% CI: 1.03–4.39; *p* = 0.041), solid organ tumor (adjusted OR 3.72; 95% CI: 1.63–8.51; *p* = 0.002), and higher body temperature (adjusted OR 1.49 per °C; 95% CI: 1.15–1.93; *p* = 0.002). The model showed good fit (Hosmer–Lemeshow *p* = 0.326). **Conclusions:** Patients presenting with hypertension, solid organ tumor, or elevated body temperature at the ED are more likely to have *E. coli* bacteremia than *S. aureus*. These predictors may support early empirical antibiotic decision-making.

## 1. Introduction

Bacteremia is considered a critical condition in the emergency department (ED). A report from a 450-bed hospital in Spain found that the incidence of bacteremia had increased from 43.8 in 1995 to 205/100,000/year in 2020 [1]. This condition had a mortality rate of 17.4% at day 30, but the mortality of bacteremia in the ICU may be higher, at 45% [2]. Antibiotic treatment is the primary management strategy for bacteremia. However, 17.71% of patients received an appropriate empirical antibiotic treatment [1,3].

*Staphylococcus aureus* and *Escherichia coli* are the two most common Gram-positive and Gram-negative bacteria associated with bacteremia [4]. The incidence of these bacteria in patients with bacteremia is exceptionally comparable, at 65 cases per 100,000 per year for *S. aureus* and up to 63.5 cases per 100,000 person years for *E*. *coli* [5,6,7]. However, *E*. *coli* was the most common cause of bacteremia, accounting for 27% of cases [7]. The mortality rate of *S. aureus* bacteremia, as reported by a systematic review of 341 studies, increased over time: 10.4% at 7 days, 18.1% at 1 month, and 30.2% at 1 year [8]. A study from England showed a comparable 30-day mortality rate of *E. coli* bacteremia of 18.2% [9]. Both studies showed that mortality from *S. aureus* and *E. coli* was high within 3 months and 14 days, respectively [8,9].

Patients with bacteremia caused by *S. aureus* and *E. coli* may present with similar symptoms, vital signs, and baseline characteristics. Patients with *S. aureus* bacteremia had an average age of 65 years, with 57% of the patients being male. In contrast, patients with *E. coli* bacteremia had a median age of 63 years, with 56.2% of the patients being male [10,11].

Although *S. aureus* and *E. coli* are both common causes of bacteremia, their initial clinical presentations often overlap. Patients typically present with nonspecific signs such as fever, hypotension, or leukocytosis, which do not indicate the underlying pathogen. This diagnostic uncertainty is particularly relevant in the emergency setting, where rapid treatment decisions are necessary but microbiological confirmation may take several hours to days. As a result, emergency physicians often must choose empirical antibiotics without knowing whether the infection is caused by a Gram-positive or Gram-negative organism, thereby risking inappropriate initial therapy. Identifying clinical factors that distinguish *E. coli* from *S. aureus* bacteremia at presentation could support more targeted empirical treatment.

However, as these two pathogens require different empirical antibiotics, it is clinically significant to evaluate whether any early clinical features can help differentiate between them while awaiting blood culture results. Additionally, there is little evidence of predictors specific to these pathogens at the ED. Therefore, this study aimed to identify clinical factors predictive of *E. coli* versus *S. aureus* bacteremia in ED patients, to aid in early risk stratification and guide empirical antimicrobial treatment decisions.

## 2. Results

A total of 327 patients met the study criteria. A total of 272 patients (83.18%) had positive cultures for *E. coli*. Among the baseline factors, the following four factors were significantly different between the *S. aureus* and *E. coli* groups: age, body temperature, and proportions of hypertension and solid organ tumor (Table 1). The *E. coli* group had a significantly higher age (67 vs. 60 years; *p* = 0.040) and body temperature (38.4 vs. 38.0 °C; *p* = 0.010) than the *S. aureus* group. The proportions of patients with hypertension (35.66% vs. 21.82%; *p* = 0.047) and solid organ tumor (32.72% vs. 14.55%; *p* = 0.007) were also higher in the *E. coli* group than the *S. aureus* group.

Regarding factors that predict *E. coli* bacteremia, four variables were included in the final model (Table 2). Of these, three were significantly associated with *E. coli* bacteremia: hypertension, solid organ tumor, and body temperature. Patients with hypertension had twice the odds of *E. coli* bacteremia compared to those without (adjusted OR: 2.12; 95% CI: 1.03–4.39), suggesting that underlying vascular or metabolic conditions may predispose individuals to Gram-negative infections. The presence of a solid organ tumor was the strongest predictor (adjusted OR: 3.72; 95% CI: 1.63–8.51), possibly reflecting increased exposure to healthcare environments, chemotherapy-induced immunosuppression, or gastrointestinal translocation. A higher body temperature was also independently associated with *E. coli* bacteremia (adjusted OR: 1.49 per °C; 95% CI: 1.15–1.93), indicating that the febrile response may be more robust in Gram-negative infections. The predictive model demonstrated good calibration (Hosmer–Lemeshow chi-square = 9.19; *p* = 0.326), supporting its reliability for clinical interpretation.

**Figure 1:**
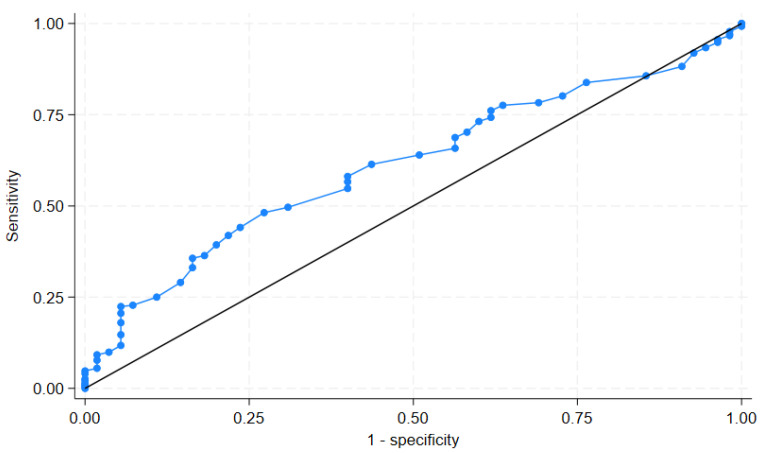
The receiver operating characteristic curve of body temperature in *Eschericia coli* bacteremia patients presenting in the emergency department.

A receiver operating characteristic (ROC) analysis of body temperature as a predictor of *E. coli* bacteremia yielded an area under the curve (AUC) of 61.07% (95% confidence interval: 53.59%, 68.55%), as shown in Figure 1. Although the discriminatory ability is modest, this suggests that an elevated body temperature may provide some value in differentiating *E. coli* from *S. aureus* bacteremia in the emergency department (ED) setting when used in conjunction with other clinical factors. While the ROC curve provides a general measurement of discrimination, we did not calculate sensitivity and specificity for specific temperature thresholds, as body temperature was not intended to be used as a standalone diagnostic marker.

The *E. coli* group had a significantly lower proportion of patients with mechanical ventilators than the *S. aureus* group (9.56% vs. 25.45%; *p* = 0.001) and a shorter length of stay (10.31 vs. 13.25 days; *p* = 0.012). Both groups had non-significant proportions of both vasopressor therapy and the other two outcomes, ICU transfer and 28-day mortality rate (Table 3). Note that the *S. aureus* group had slightly higher proportions of vasopressor therapy (36.36% vs. 30.51%; *p* = 0.394), ICU transfer (41.82% vs. 36.39%; *p* = 0.448), and mortality rate (12.73% vs. 7.72%; *p* = 0.286) than the *E. coli* group.

## 3. Discussion

This study identified three independent factors associated with *E. coli* bacteremia in the ED: hypertension, solid organ tumor, and elevated body temperature.

The finding that hypertension is independently associated with *E. coli* bacteremia may reflect underlying vascular, renal, or immune factors that predispose hypertensive patients to Gram-negative infections. Although the mechanism is unclear, the clinical implications are essential. In patients presenting to the ED with sepsis and a history of hypertension, clinicians may consider a higher likelihood of *E. coli* as the causative pathogen. This may support early empirical coverage targeting Gram-negative organisms in such patients, pending confirmation of culture results.

*E. coli* was the common pathogen in patients with solid organ tumor, as these patients may have a higher risk of gastrointestinal enterocolitis [12]. Additionally, a study conducted in Spain found that 40.8% of patients with solid organ cancer and bacteremia received antibiotics frequently [13]. This may cause Gram-negative bacteremia, particularly resistant *E. coli* infection. Finally, patients with solid organ tumor may receive chemotherapy and develop *E. coli* bacteremia while in a neutropenic state [14,15].

A study from China found that several parameters were associated with Gram-negative bacteremia at the ED, including body temperature, respiratory rate, Glasgow coma scale, heart rate, qSOFA, and SIRS [16]. This study found that only body temperature was associated with *E. coli* bacteremia (Table 2). Differences in pathogens and the sources of cultures may account for these variations. The previous study included any Gram-negative bacteria and several sources of cultures, such as blood, sputum, and urine. Note that blood cultures accounted for approximately 25% of the patients with *E. coli* infection [16]. In contrast, this study was specifically on *E. coli* bacteremia. Among the significant factors identified in the previous study, only body temperature was found to be substantial in this study. These findings suggest that a higher body temperature may be more commonly associated with *E. coli* bacteremia. Still, it should not be used alone to exclude *S. aureus* as a possible pathogen. A previous study supports these findings as it showed that an increase in cytokines such as interleukin-2, 6, 8, and interferon gamma was associated with an increase in temperature to 39 °C [17].

Although age was significantly related to *E. coli* bacteremia in univariable logistic regression analysis, this relationship was not significant in the multivariable logistic regression model (Table 2). These findings may suggest that age may not be an important factor compared to other factors. A previous study from England even found that *E. coli* bacteremia was seen more in patients aged 65 years or older, at 70.5% [6].

This study has some limitations. First, the setting is a single tertiary care hospital with a focus on community-acquired infections, which may limit the generalizability of the findings to other regions or healthcare systems. Second, the predictive model was based solely on clinical variables available at presentation and did not incorporate laboratory or imaging data, which may have improved its performance. Third, molecular diagnostic tools such as PCR or pathogen-specific biomarkers were not utilized, which limited the ability to detect mixed or fastidious infections. Finally, the retrospective design may be prone to selection or documentation bias, and specific comorbidities or clinical nuances may not have been fully captured in the chart review process.

## 4. Materials and Methods

This was a retrospective analytical study conducted at the Department of Emergency Medicine, Faculty of Medicine, Khon Kaen University, Thailand. A retrospective design was chosen to assess real-world clinical and microbiological data from existing records. It enabled the efficient identification of predictors based on actual presentations of bacteremia in the emergency setting, without introducing selection bias through prospective screening.

The inclusion criteria were adult patients aged 18 years or older presenting with bacteremia in the ED and having blood culture results positive for either *S. aureus* or *E. coli*. Pregnant women, patients who had received antibiotic treatment during the current illness episode before ED presentation, and those with other causative organisms were excluded. The study period was between 1 January 2016 and 31 December 2018. The study protocol was approved by the Ethics Committee for Human Research at Khon Kaen University, Thailand (HE661442). This study was a part of the blood culture project taking place in the emergency department and was previously published in an article [3].

Sepsis was defined as the presence of clinical signs of systemic infection at emergency department (ED) presentation, including features such as fever, hypotension, leukocytosis, altered mental status, or organ dysfunction. These findings were assessed using criteria aligned with the systemic inflammatory response syndrome (SIRS) or the quick Sequential Organ Failure Assessment (qSOFA) score, based on the practices in place at the time of data collection. Bacteremia was confirmed by a positive blood culture for either *E. coli* or *S. aureus* in patients with suspected sepsis.

A retrospective medical chart review was performed in eligible patients. Baseline characteristics, vital signs, management, and treatment outcomes were recorded. Data on comorbidities, Charlson comorbidity index scores, SIRS, qSOFA, National Early Warning Scores (NEWs), and oxygen saturation were collected. The Charlson comorbidity index comprises 17 factors used to predict the 10-year survival rate in patients with multiple comorbidities [18]. These factors are age, myocardial infarction, congestive heart failure, peripheral vascular disease, cerebrovascular accident or transient ischemic attacks, dementia, chronic pulmonary disease, connective tissue disease, peptic ulcer disease, liver disease, diabetes mellitus, hemiplegia, moderate to severe chronic kidney disease, solid tumor, leukemia, lymphoma, and AIDS. A Charlson comorbidity index score of zero indicates no comorbidity, while a higher score indicates comorbidities associated with a higher mortality rate.

The sample size was based on all eligible cases identified during the 3-year study period. A formal a priori sample size calculation was not performed, as this was an exploratory study aiming to include the full population of adult ED patients with blood culture-confirmed *E. coli* or *S. aureus* bacteremia. This approach ensured adequate statistical power based on available real-world data.

The outcomes of treatment included ICU transfer, length of stay, and 28-day mortality rate, retrieved from medical chart records of follow-ups. The management of mechanical ventilator and vasopressor therapy was also recorded.

For statistical analysis, patients were categorized into two groups: those with *S. aureus* and those with *E. coli*, based on the results of the blood culture. Descriptive statistics were used to describe the characteristics of the studied variables, while inferential statistics were used to compare the differences between the variables studied in the two groups. The Wilcoxon rank sum test and Fisher’s Exact test were used to compare the differences in median and proportion between the two groups, respectively. Clinical factors predictive of *E. coli* bacteremia were identified by multivariable logistic regression analysis. Univariable logistic regression analysis was performed. The significant factors identified by univariable logistic regression analysis were included in the subsequent multivariable logistic regression analysis. The predictive model was tested for goodness of fit by the Hosmer–Lemeshow method. A receiver operating characteristic (ROC) curve was generated to evaluate the discriminatory performance of body temperature, a continuous variable found to be significantly associated with *E. coli* bacteremia in the multivariable model. The ROC curve plots the true positive rate (sensitivity) against the false positive rate (1—specificity) across a range of temperature thresholds. The area under the curve (AUC) was calculated to quantify the overall ability of body temperature to distinguish *E. coli* from *S. aureus* bacteremia. The AUC values ranged from 0.5 (no discrimination) to 1.0 (perfect discrimination). Specific cut-off points were not determined, as the analysis was intended to assess general discriminatory performance rather than develop a diagnostic threshold. All statistical analyses were performed using STATA software (version 18.0, College Station, TX, USA).

## 5. Conclusions

Patients presenting to the emergency department with hypertension, solid organ tumor, or an elevated body temperature were more likely to have *E. coli* bacteremia compared to *S. aureus*. These clinical characteristics may help inform early risk assessment while awaiting confirmation of blood culture results. However, as this was a single-center retrospective study, the findings should be interpreted with caution. Future prospective multicenter studies are needed to validate these predictors and to assess their utility in guiding empirical antibiotic selection in real-world emergency care settings. Patients presenting with hypertension, solid organ tumor, or an elevated body temperature in the ED were more likely to have *E. coli* bacteremia compared to *S. aureus*. These clinical predictors may help guide early empirical antibiotic selection in the emergency setting.

## Figures and Tables

**Table 1:** The baseline characteristics of patients presenting in the emergency department with bacteremia caused by *Staphylococcus aureus* or *Escherichia coli*.

Factor	*S. aureus* n = 55	*E. coli* n = 272	*p* Value
Age, years	60 (52, 72)	67 (56, 77)	0.040
Male sex	34 (61.82)	138 (50.74)	0.133
Comorbidities			
Hypertension	12 (21.82)	97 (35.66)	0.047
Diabetes	19 (34.55)	65 (23.90)	0.127
Chronic kidney disease	10 (18.18)	28 (10.29)	0.096
Stroke	3 (5.45)	11 (4.04)	0.713
Solid organ tumor	8 (14.55)	89 (32.72)	0.007
Charlson comorbidity index score	3 (1, 5)	4 (2, 5)	0.057
SIRS	3 (2, 3)	3 (2, 3)	0.803
qSOFA	1 (1, 2)	1 (1, 1)	0.247
NEW	6 (4, 8)	5 (4, 8)	0.733
Body temperature, °C	38.0 (37.0, 38.6)	38.4 (37.4, 39.4)	0.010
Pulse rate, bpm	98 (90, 100)	96 (89, 107)	0.552
Respiratory rate, tpm	27 (22, 32)	24 (22, 30)	0.218
Systolic blood pressure, mmHg	117 (96, 138)	125 (103, 145)	0.111
Diastolic blood pressure, mmHg	69 (58, 80)	70 (60, 82)	0.543
Oxygen saturation, %	97 (95, 99)	97 (95, 99)	0.733

Data are presented as a number (percentage) or median (1st–3rd interquartile range). SIRS: systemic inflammatory response syndrome; qSOFA: quick sepsis-related organ failure assessment; NEW: National Early Warning Score; bpm: beats per minute; tpm: times per minute; mmHg: millimeters of mercury.

**Table 2:** The factors predictive of *Escherichia coli* bacteremia presented in the emergency department.

Factor	Unadjusted Odds Ratio (95% Confidence Interval); *p* Value	Adjusted Odds Ratio (95% Confidence Interval); *p* Value
Age, year	1.02 (1.01, 1.04); 0.030	1.01 (0.99, 1.03); 0.083
Hypertension	1.98 (0.99, 3.94); 0.050	2.12 (1.03, 4.39); 0.041
Solid organ tumor	2.85 (1.29, 6.30); 0.009	3.72 (1.63, 8.51); 0.002
Body temperature, °C	1.37 (1.08, 1.75); 0.010	1.49 (1.15, 1.93); 0.002

**Table 3:** The management and outcomes of patients presenting in the emergency department with bacteremia caused by *Staphylococcus aureus* or *Escherichia coli*.

Factor	*S. aureus* n = 55	*E. coli* n = 272	*p* Value
Mechanical ventilator	14 (25.45)	26 (9.56)	0.001
Vasopressor therapy	20 (36.36)	83 (30.51)	0.394
ICU transfer	23 (41.82)	99 (36.39)	0.448
Length of stay, days	13.25 (8.01, 24.21)	10.31 (6.83, 15.68)	0.012
Mortality at 28th day	7 (12.73)	21 (7.72)	0.286

Data are presented as a number (percentage) or median (1st–3rd interquartile range).

## Data Availability

The data are available from the corresponding author upon reasonable request.

## References

[1] García-Rodríguez J.F., Mariño-Callejo A. (2023). The Factors Associated with the Trend in Incidence of Bacteraemia and Associated Mortality over 30 Years. BMC Infect. Dis..

[2] Nasa P., Juneja D., Singh O., Dang R., Arora V., Saxena S. (2011). Incidence of Bacteremia at the Time of ICU Admission and Its Impact on Outcome. Indian. J. Anaesth..

[3] Phungoen P., Kraisriwattana A., Apiratwarakul K., Wonglakorn L., Sawanyawisuth K. (2019). Predictors of Appropriate Antibiotic Use in Bacteremia Patients Presenting at the Emergency Department. Antibiotics.

[4] Poolman J.T., Anderson A.S. (2018). *Escherichia coli* and *Staphylococcus aureus*: Leading Bacterial Pathogens of Healthcare Associated Infections and Bacteremia in Older-Age Populations. Expert. Rev. Vaccines.

[5] Hindy J.-R., Quintero-Martinez J.A., Lee A.T., Scott C.G., Gerberi D.J., Mahmood M., DeSimone D.C., Baddour L.M. (2022). Incidence Trends and Epidemiology of *Staphylococcus aureus* Bacteremia: A Systematic Review of Population-Based Studies. Cureus.

[6] Bou-Antoun S., Davies J., Guy R., Johnson A.P., Sheridan E.A., Hope R.J. (2016). Descriptive Epidemiology of *Escherichia coli* Bacteraemia in England, April 2012 to March 2014. Eurosurveillance.

[7] Bonten M., Johnson J.R., van den Biggelaar A.H.J., Georgalis L., Geurtsen J., de Palacios P.I., Gravenstein S., Verstraeten T., Hermans P., Poolman J.T. (2021). Epidemiology of *Escherichia coli* Bacteremia: A Systematic Literature Review. Clin. Infect. Dis..

[8] Bai A.D., Lo C.K.L., Komorowski A.S., Suresh M., Guo K., Garg A., Tandon P., Senecal J., Del Corpo O., Stefanova I. (2022). *Staphylococcus aureus* Bacteraemia Mortality: A Systematic Review and Meta-Analysis. Clin. Microbiol. Infect..

[9] Abernethy J.K., Johnson A.P., Guy R., Hinton N., Sheridan E.A., Hope R.J. (2015). Thirty Day All-Cause Mortality in Patients with *Escherichia coli* Bacteraemia in England. Clin. Microbiol. Infect..

[10] Daga A.P., Koga V.L., Soncini J.G.M., de Matos C.M., Perugini M.R.E., Pelisson M., Kobayashi R.K.T., Vespero E.C. (2019). *Escherichia coli* Bloodstream Infections in Patients at a University Hospital: Virulence Factors and Clinical Characteristics. Front. Cell Infect. Microbiol..

[11] Yanık-Yalçın T., Erol Ç., Demirkaya M.H., Durukan E., Kurt-Azap Ö. (2023). Evaluation of Clinical Approach and Outcomes *Staphylococcus aureus* Bacteremia. Infect. Dis. Clin. Microbiol..

[12] Rolston K.V.I. (2017). Infections in Cancer Patients with Solid Tumors: A Review. Infect. Dis. Ther..

[13] de Dios-García B., Maestro G., Díaz-Pedroche C., Parra W., Campos Ó., Orellana M.Á., Caro J.M., Lumbreras C., Lizasoain M. (2023). Bacteremia in Patients with Solid Organ Cancer: Insights into Epidemiology and Antibiotic Consumption. Cancers.

[14] Gustinetti G., Mikulska M. (2016). Bloodstream Infections in Neutropenic Cancer Patients: A Practical Update. Virulence.

[15] John K.R., Warrier A., Warrier A. (2023). Microbiological Spectrum of Neutropenic Sepsis in Cancer Patients Admitted to a Tertiary Health Care Centre. Cureus.

[16] Hsu C.-P., Chen H.-Y., Chen W.-L., Chen J.-H., Huang C.-C., Wu P.-H., Chung J.-Y. (2021). Clinical Physiological Parameters for the Prediction of Gram-Negative Bacterial Infection in the Emergency Department. BMC Infect. Dis..

[17] Chaban V., de Boer E., McAdam K.E., Vaage J., Mollnes T.E., Nilsson P.H., Pischke S.E., Islam R. (2023). *Escherichia coli*-Induced Inflammatory Responses Are Temperature-Dependent in Human Whole Blood Ex Vivo. Mol. Immunol..

[18] Charlson M.E., Carrozzino D., Guidi J., Patierno C. (2022). Charlson Comorbidity Index: A Critical Review of Clinimetric Properties. Psychother. Psychosom..

